# Characteristics of physical activity during beginner-level group tennis lessons and the effect daily activity

**DOI:** 10.1038/s41598-023-46843-0

**Published:** 2024-01-02

**Authors:** Ryo Kawakami, Satoshi Yamakawa, Shoji Konda, Issei Ogasawara, Ryosuke Hasegawa, Keita Yamasaki, Takashi Kanamoto, Teruki Yokoyama, Akiyo Yoshida, Yoshihiro Marutani, Yuko Ueda, Hideo Mitsuoka, Shintaro Horie, Daigo Horio, Ken Nakata

**Affiliations:** 1 Department of Health and Sport Sciences, Osaka University Graduate School of Medicine, Toyonaka, Osaka 560-0043 Japan; 2 Department of Sports Medical Biomechanics, Osaka University Graduate School of Medicine, Suita, Osaka 565-0871 Japan; 3 Graduate School of Sport Science, Osaka University of Health and Sports Sciences, Kumatori, Osaka 590-0496 Japan; 4 GODAI Incorporated Educational Institution, Yokohama, Kanagawa 220-6220 Japan

**Keywords:** Health care, Health occupations

## Abstract

Tennis is a popular leisure sport, and studies have indicated that playing tennis regularly provides many health benefits. We aimed to clarify the characteristics of physical activity during beginner-level group tennis lessons and daily physical activity of the participants. Physical activity was measured using an accelerometer sensor device for four weeks, including the 80-min duration tennis lessons held twice a week. Valid data were categorized for tennis and non-tennis days. The mean physical activity intensity during the tennis lesson was 3.37 METs. The mean ratio of short-bout rest periods to the tennis lesson time in 90 and 120 s was 7% and 4%, respectively. The mean physical activity intensity was significantly higher (*p* < 0.0001) and the duration of vigorous-intensity physical activity (VPA) was increased in 76% of participants on days with tennis lessons compared to without tennis lessons. Beginner-level tennis lesson has characteristics of less short-bout rest physical activity than previously reported competitive tennis match and increased the duration of VPA in daily activity compared to without tennis lessons, suggesting that beginner-level tennis lessons contribute physical activity of health benefits.

## Introduction

Physical inactivity is a well-known risk factor for the development of various diseases, and solving the problem of physical inactivity contributes to an increase in life expectancy^1^. As a guideline to increase physical activity, for example, the World Health Organization (WHO) recommends that “adults should undertake 150–300 min of moderate-intensity aerobic physical activity or 75–150 min of vigorous-intensity aerobic physical activity per week”^2^. In addition, the duration of continuous physical activity and physical activity type have been investigated. The beneficial effects of intermittent short-bout moderate-to-vigorous physical activity (MVPA), defined as less than 10 min duration as well as long duration, have been shown to reduce the risk of cardiovascular disease and death^3–5^.

There are factors that lead to physical inactivity, such as an environment that limits opportunities for walking and reduces occupational activity^6^. The WHO recommends increasing steps, exercising, and playing sports daily as a solution to physical inactivity^2^. Tennis is one of the most popular leisure sports for people of all ages, from competitive to recreational levels, worldwide^7,8^. The previous study showed that well-experienced and regularly playing tennis players meet several positive effects, including enhanced aerobic capacity, increased bone density, reduced body fat, elevated muscle strength, and a decrease in cognitive decline^9^. In addition, a longitudinal study followed over a period of 22 to 40 years on male college students majoring in tennis has shown that continuous engagement in tennis significantly improved cardiopulmonary function and demonstrated a reduction in the risk of developing conditions such as diabetes and cardiovascular disease^10^. Joining a community to play tennis can also prevent cardiovascular disease by promoting social interaction, ongoing physical activity, and reducing loneliness and stress^9^.

Various studies have reported the characteristics of physical activity during a tennis match in competitive-level players^11–16^. In general, a tennis match consists of a repetition of vigorous-intensity physical activity interspersed with rest periods of defined duration (10–20 s between points, 90 s during court changes, and 120 s between sets)^11,12^. Actual playing time, which excludes rest time from match time, has been reported to be 10–30% of the match time^12,13^. In addition, although the duration is separated by 2–12 s between each point, there are different characteristics from continuous exercise^12,14,15^. Previous studies have shown that physical activity intensity during tennis is classified as moderate-to-vigorous based on oxygen intake measured during matches and practices^8,11,13,16^. Also, the Internationally used Compendium of Physical Activity doses states that playing tennis is equivalent to 4.5–8 METs^17^.

Previous studies have mainly focused on young competitive-level players who practiced tennis intensively^12,18–20^. There are no studies that have focused on physical activity during tennis on beginner-level and alteration of physical activity in daily life. However, beginner-level tennis players may not achieve the same intensity of physical activity as competitive-level tennis players due to their lower skill level in comparison to competitive-level tennis players. Therefore, it is necessary to observe physical activity during tennis at the beginner-level and in daily life, and to examine whether playing tennis at the beginning-level can lead to the acquisition of healthy physical activity.

Participating in a lesson at a tennis school is an option for recreational players who wish to habitually play tennis. Tennis schools generally conduct group lessons (referred to as -tennis lessons-), for 5–10 players. Previous studies have frequently focused on the physical activity intensity of singles and doubles matches played by two or four players per court, respectively^12–15,21^. However, the physical activity intensity and characteristics of tennis lessons in groups of 5–10 players per tennis court have not yet been clarified. In addition, it is still unclear whether attending tennis schools provides health benefits. Therefore, we aimed to clarify the characteristics of physical activity during tennis lessons and in daily life among beginner-level tennis lesson participants.

## Results

**Figure 1:**
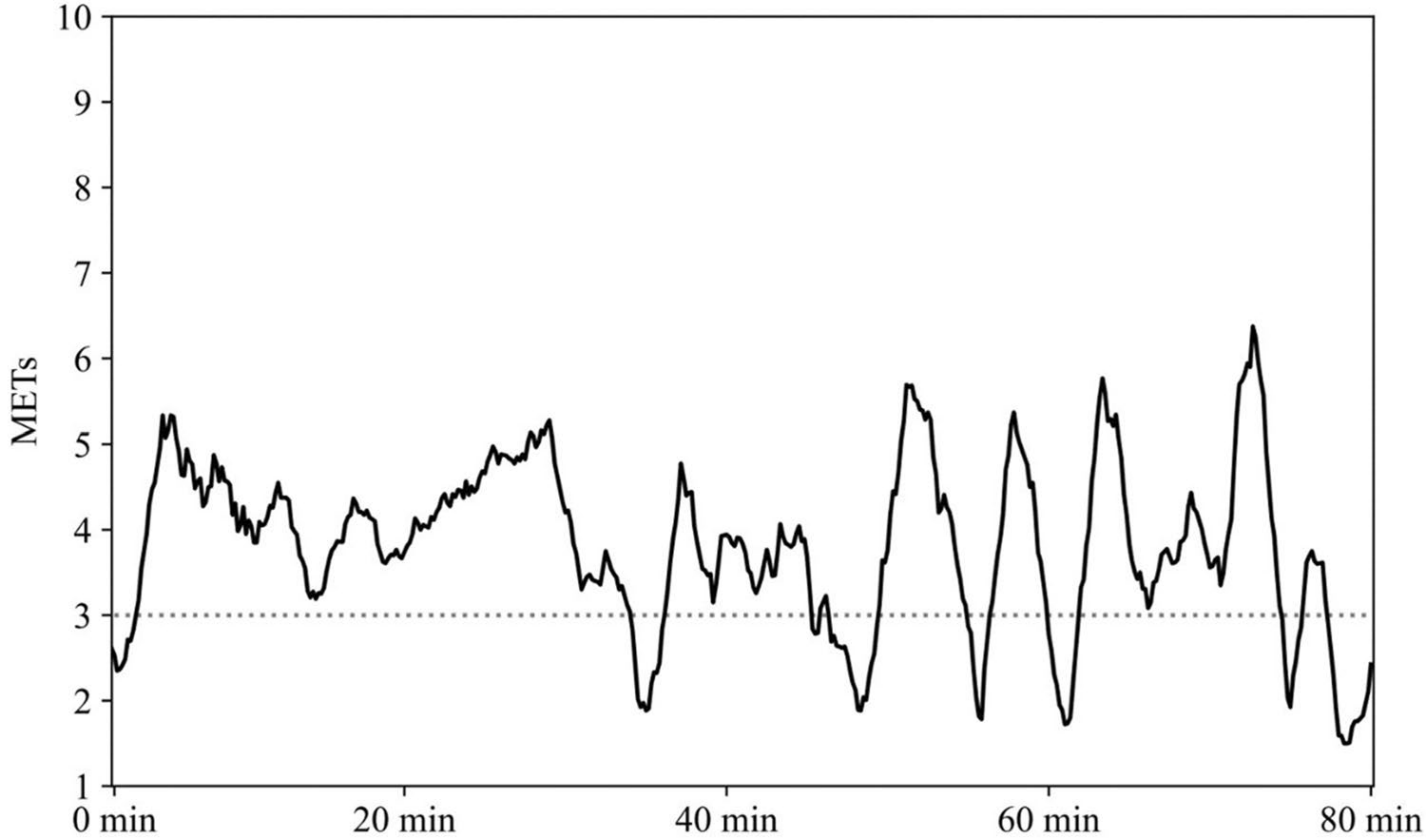
A typical waveform of the physical activity intensity during the tennis lesson. Gray dotted line indicates 3 METs, which is the lower threshold of the MVPA.

**Figure 2:**
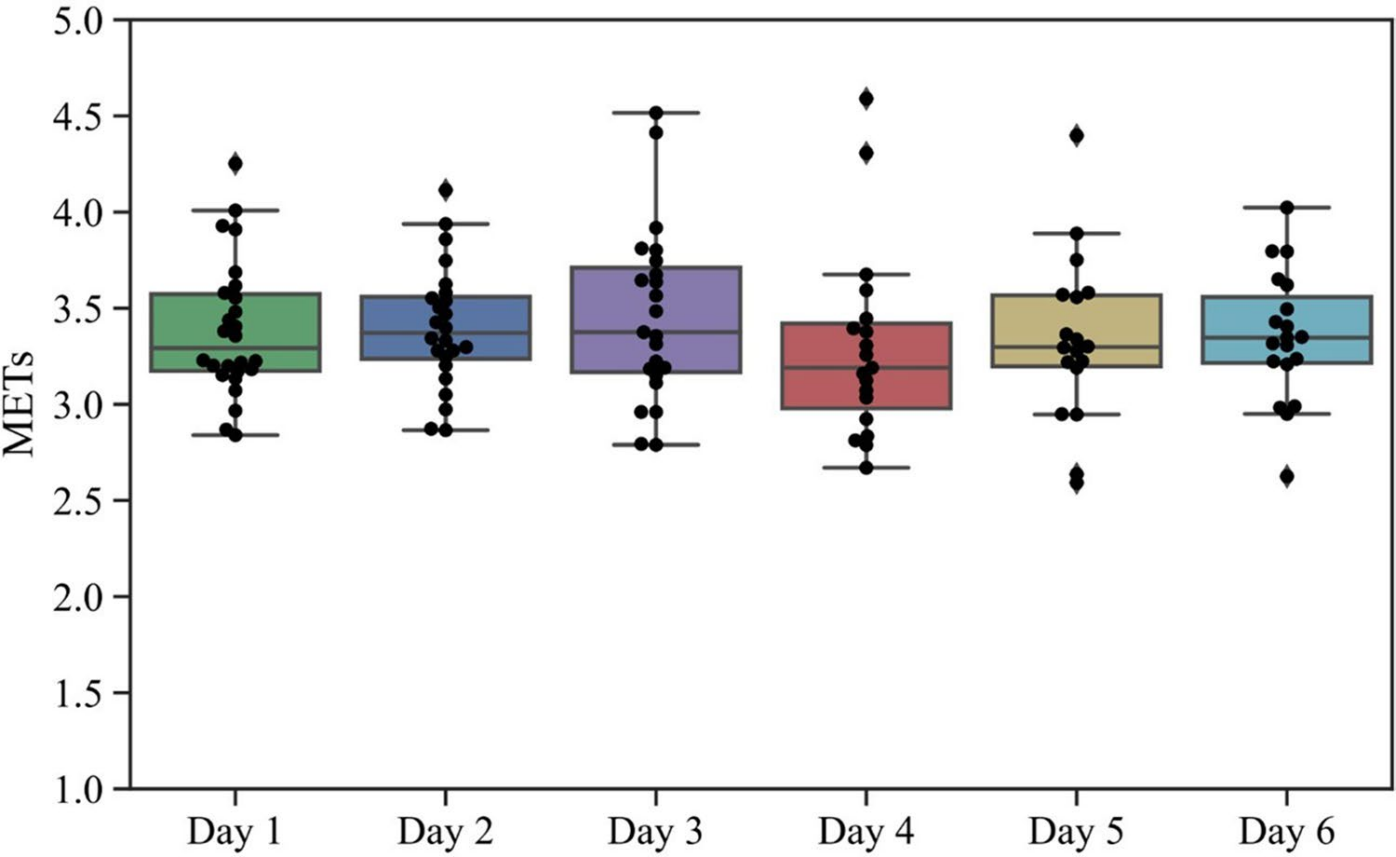
Mean intensity of physical activity during daily tennis lesson.

Valid accelerometer data were obtained from the 26 participants (Table 1) for 538 days (129 tennis days; and 409 non-tennis days). The typical time-series data of physical activity intensity during the tennis lessons are shown in Fig. 1. The mean physical activity intensity during the tennis lesson was 3.37 ± 1.64 METs. The physical activity intensity during each tennis lessons during the measurement periods showed no significant differences from day to day (*F* [5, 123] = 0.44, *p* = 0.82, Fig. 2). The mean ratio of short-bout rest periods compared to tennis lesson time was 7% and 4% for 90 and 120 s rest periods, respectively (Table 2).

**Table 1:** Characteristics of subjects.

Characteristics	
N, (men)	26 (5)
Age, years, mean (SD)	55.6 ± 14
Height, cm, mean (SD)	159.5 ± 5.1
Weight, kg, mean (SD)	54.1 ± 8.9
Body mass index, kg/m^2^, mean (SD)	21.2 ± 3.1
Tennis Experience, years, median (IQR)	3.5 (1–8)

*SD* standard deviation, *IQR* interquartile range.

**Table 2:** Variables during tennis lessons.

Variables	During tennis lesson
METs, METs/times, mean (SD)	3.37 ± 1.64
Short-bout length, %/times, mean (SD)	
90 s	7 ± 0.03
120 s	4 ± 0.03

*METs* metabolic equivalents, *SD* standard deviation.

**Figure 3:**
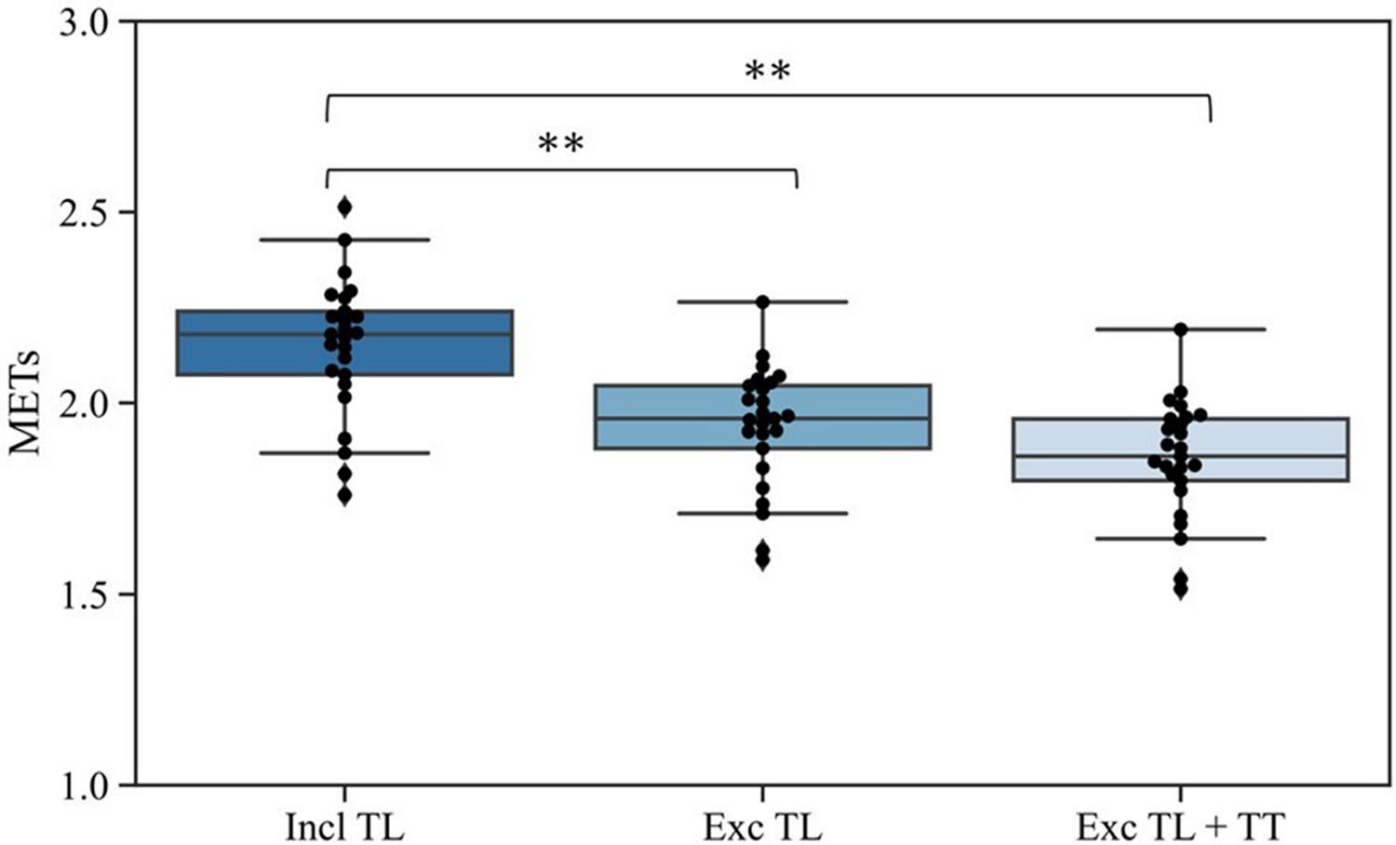
Mean intensity of physical activity on tennis lesson days. Incl TL, Mean intensity of physical activity per day on whole the day of tennis; Exc TL, mean intensity of physical activity per day excluding tennis lessons from tennis days. Exc TL + TT, mean intensity of physical activity per day, excluding during tennis lessons and travel times from tennis days. **p < 0.001.

The mean physical activity intensity on the days performed tennis lessons showed a significantly higher intensity compared to the days performed tennis lessons excluding the time during the tennis lessons, and compared to the days performed tennis lessons excluding the time during the tennis lessons and travel time (*p* < 0.001, and, *p* < 0.001, respectively, Fig. 3).

**Figure 4:**
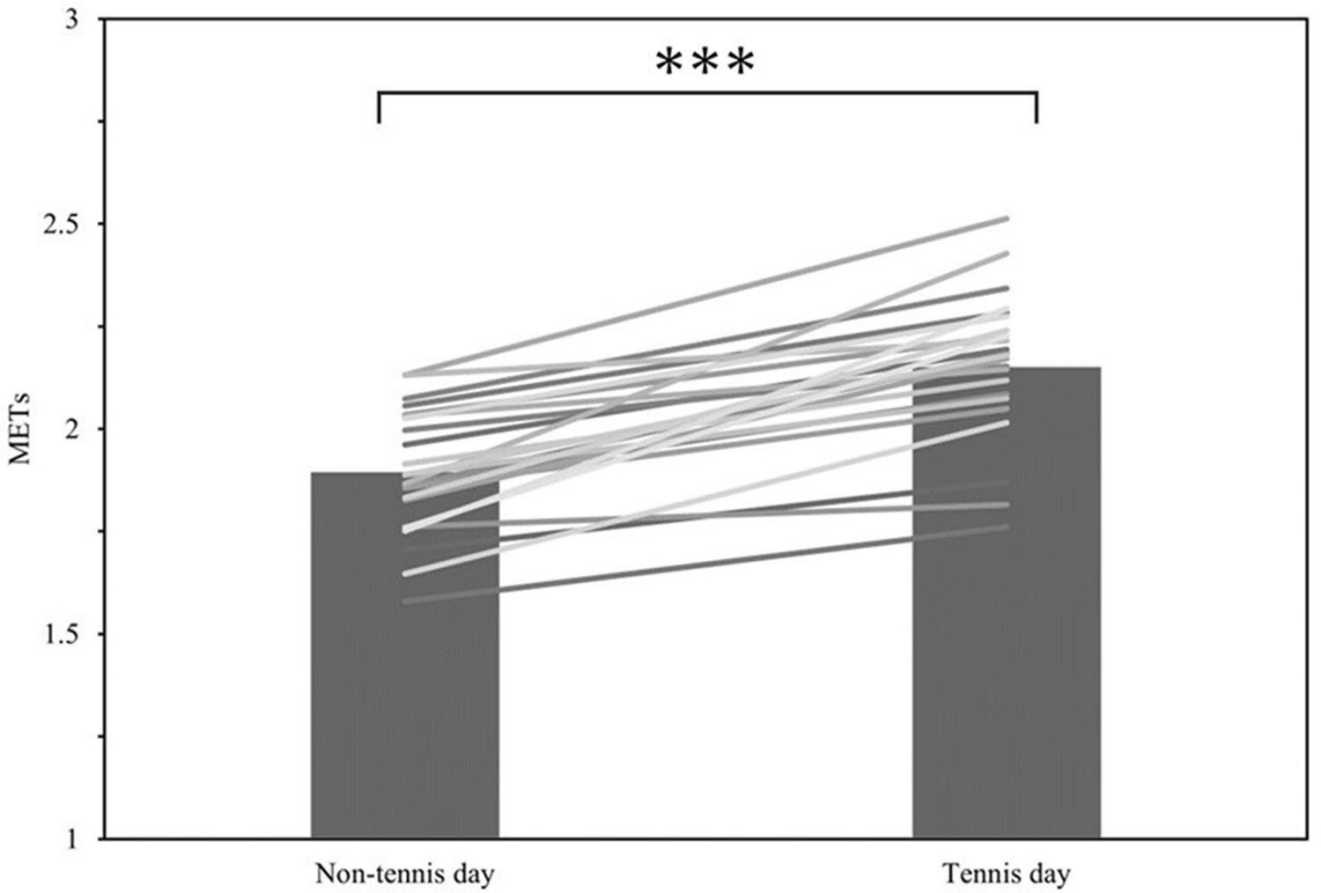
Mean intensity of physical activity per day on tennis days and non-tennis days. ***p < 0.0001.

**Figure 5:**
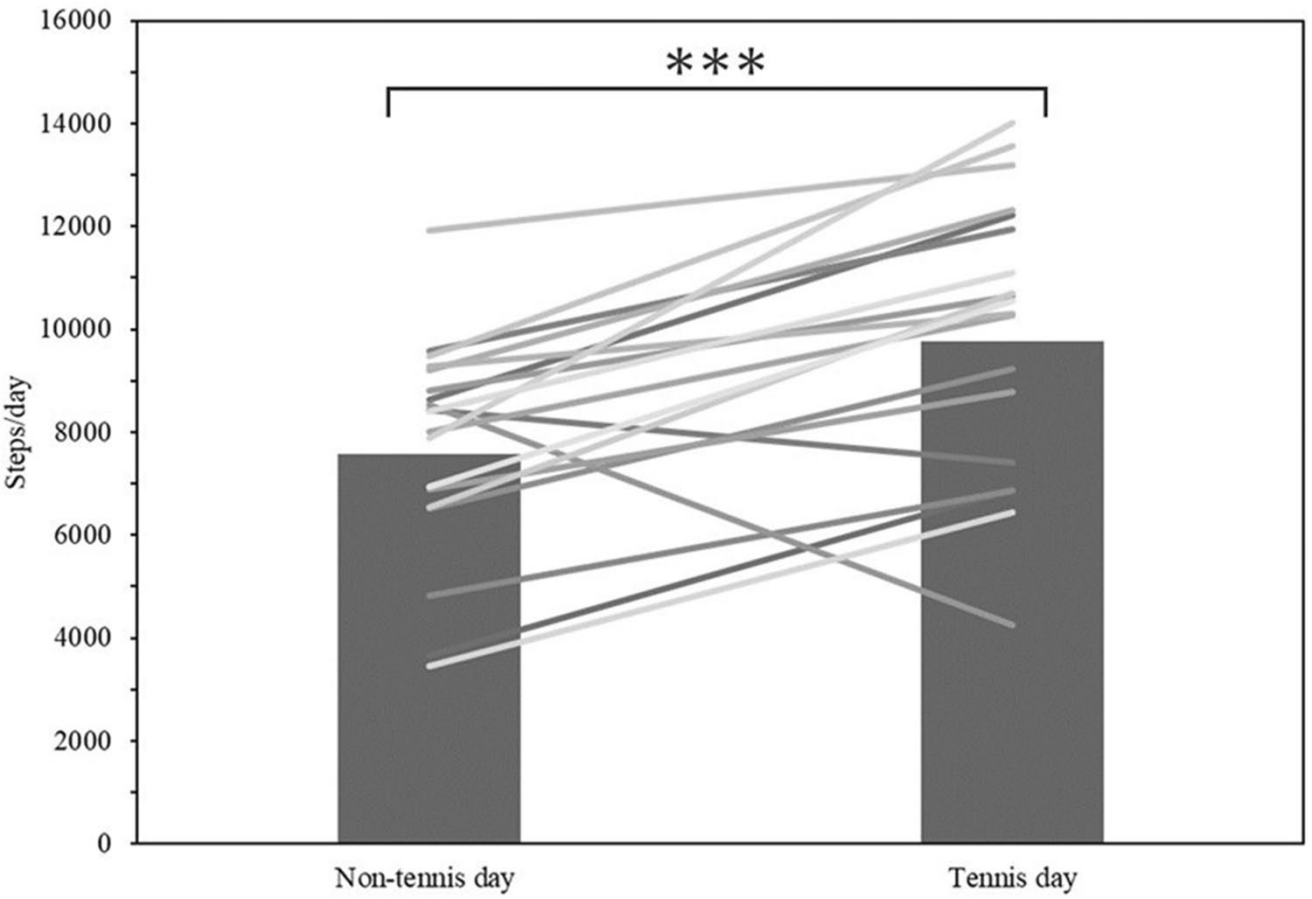
Mean number of steps per day on tennis days and non-tennis days. ***p < 0.0001.

**Figure 6:**
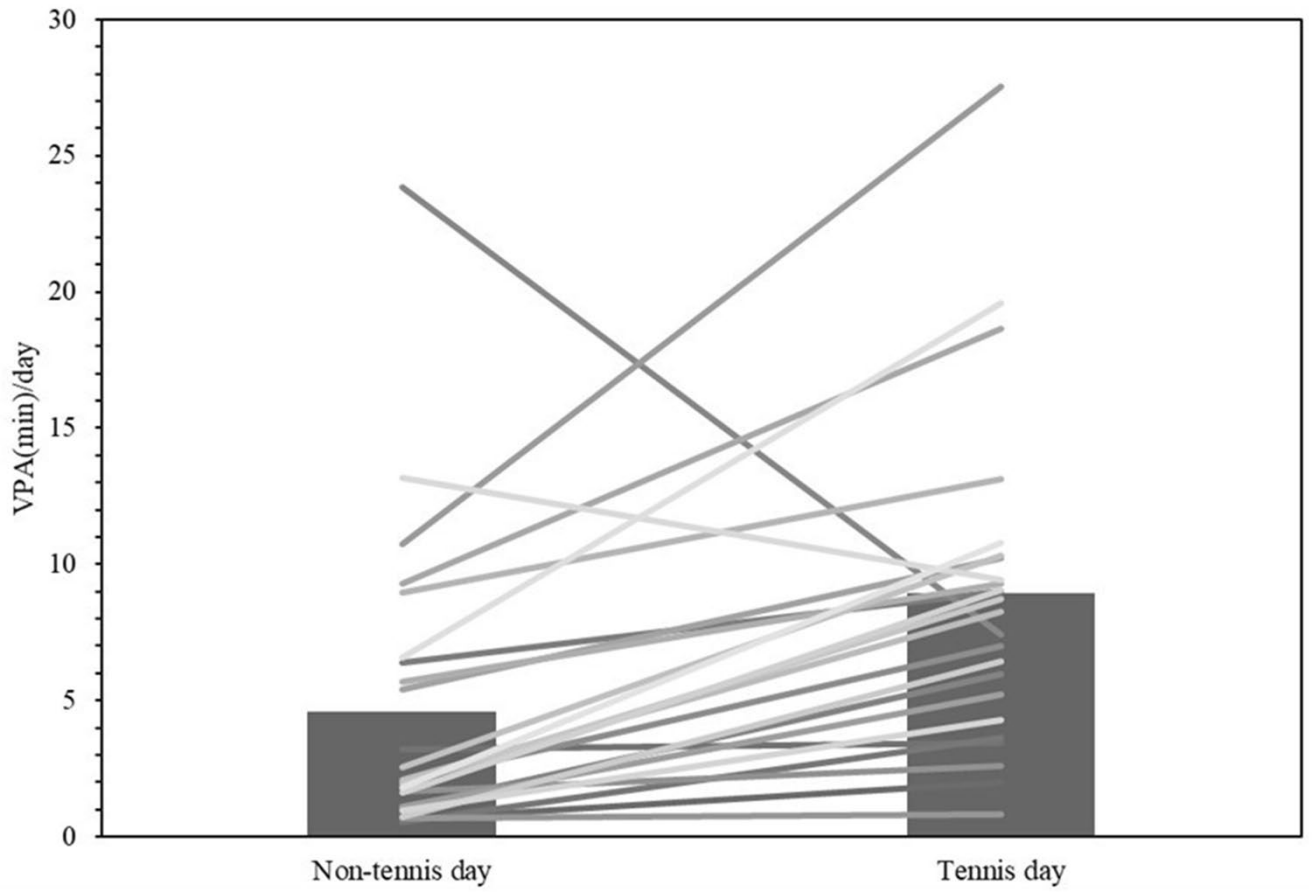
Mean vigorous physical activity time on tennis days and non-tennis lesson days.

Compared to the days of tennis lessons, the mean physical activity intensity and number of steps taken were significantly lower on the days without tennis (*p* < 0.0001 and, *p* < 0.0001, respectively) (Table 3, Figs. 4, 5). Furthermore, vigorous-intensity physical activity time increased on the days when tennis was performed compared to those it was not in 92% of subjects. Also, 76% of subjects had more than 1.5 times as many hours of vigorous-intensity physical activity on the day of tennis lessons than on the days when they did not play tennis (Fig. 6).

**Table 3:** Variables for performed tennis and unperformed tennis day: steps and intensity of physical activity (METs) per day.

Variables	All	Tennis day	Non-tennis day
Step count, steps/d, mean (SD)	7878.7 ± 1954	9772.8 ± 2612.3	7579.4 ± 2034.03
METs, METs/d, mean (SD)	1.9 ± 0.15	2.2 ± 0.18	1.9 ± 0.16
Accelerometer wear time, min/d, mean (SD)	767.5 ± 104.6		772.1 ± 110.9
SB, min/d, mean (SD)	432.6 ± 110.4	244.8 ± 78.2	371.5 ± 64.5
LPA, min/d, mean (SD)	327.4 ± 73.7	204.4 ± 41.3	282.7 ± 57.4
MPA, min/d, mean (SD)	114.9 ± 31.9	107.6 ± 20.4	94.6 ± 24.3
VPA, min/d, mean (SD)	5.7 ± 5.3	9.0 ± 5.8	4.6 ± 5.4

METs Metabolic Equivalents, SD Standard Deviation.

## Discussion

The main findings of this study, which assessed physical activity during beginner-level tennis lessons and during activities of daily living, were that participation in tennis lessons increased average daily physical activity intensity, as well as the number of steps taken and the duration of high-intensity physical activity.

The mean physical activity intensity during the tennis group lesson was lower than that during competition-level tennis matches shown in previous studies. On the other hand, our results indicated that the beginner level tennis group lesson showed continuous moderate-to-vigorous-intensity physical activities with no certain duration of rest during the lesson time, while a previous report indicated that competitive-level matches showed intermittent vigorous-intensity physical activities. These results indicate the differences in characteristics between group lessons and the tennis matches. In addition, the intensity of physical activity on the day of the tennis lesson was increased by the lesson itself. Physical activity on the day of the tennis lesson was significantly higher than that of the days without tennis lessons. Additionally, regarding the duration of vigorous-physical-activity, 76% of total participants showed a > 50% increase on the performed tennis lessons days compared to the other days.

Physical activity intensity during tennis group lessons for beginners averaged 3.37 METs in a previous study. A similar magnitude of intensity was obtained in each lesson, which means that tennis lessons provided constantly similar physical activity levels each time. In the present study, the measurements were performed with 6–12 players per group, which may have led to fewer hit and travel distances compared to the environment in previous studies. This might be the cause of the lower intensity compared to the previously reported tennis matches of 4.5–8 METs^17^. Contrarily, the activity during a tennis match is in the form of intermittent short-duration exercise, with periodic 90–120 s breaks, and the effective playing time is approximately 20% of the total activity time^12,13^. In contrast, tennis lessons showed continuous moderate-to-vigorous-intensity physical activity with little continuous rest. These findings indicate that there is a fundamental difference between physical activity during competition and tennis lessons: a short duration of vigorous-intensity physical activity or long duration of moderate-to-vigorous-intensity physical activity.

The physical activity intensity on the entire day attending lessons was significantly higher than that on days when the lesson time and both the tennis lesson time and travel time were excluded. This result indicates that tennis lessons provide a higher intensity of physical activity than the usual daily physical activity for the participants. It is suggested that activity during tennis lessons is an unusual intensity for participants in daily life, and attending the lesson contributes to increasing physical activity more than moderate-intensity physical activity. The mean daily physical activity intensity and the number of steps were significantly greater on the performed tennis lesson days than on those when tennis was not played suggesting that taking tennis lessons increased the mean daily physical activity intensity and contributed to an increase in total activity. The Ministry of Health, Labor and Welfare (MHLW) report that the mean number of steps per day was 7864 for men and 6685 for women^22^. In the present study, the mean number of steps was 7579 on the days without tennis lessons, which is almost the same as the average of the results surveyed by the MHLW, while on the days of tennis lessons, 9773 steps were taken, which is significantly higher. The health benefits of increasing the number of daily steps have been reported in previous studies, and can contribute to an increase in hours of moderate-to-vigorous-intensity physical activity^23^ and reduce risk factors for mortality of noncommunicable diseases^24,25^. These results suggest that taking tennis at the beginner level may provide health benefits by increasing mean daily physical activity intensity and number of daily steps.

Regarding the duration of the vigorous-physical-activity, 76% of total participants showed a > 50% increase in the duration on the tennis lesson days and a significantly higher duration compared to the non-tennis days. The WHO recommendation for physical activity states that adults should engage in at least 150 min of moderate-intensity physical activity, 75 min of vigorous-intensity physical activity, or a combination of moderate-and vigorous-intensity physical activity per week^2^. In addition, previous studies have stated that short-bout vigorous-intensity physical activity may have similar or greater health benefits than moderate-intensity physical activity, especially because of greater improvements in cardiorespiratory function compared with long-bout moderate-intensity physical activity^26^. Furthermore, vigorous-intensity physical activity is better when performed as much as possible^5^. Therefore, it is suggested that taking tennis lessons may provide health benefits, such as improved cardiopulmonary function, by increasing the duration of vigorous-intensity physical activity.

The present study has some limitations. First, the METs obtained were estimated values based on the accelerometers, and it needs to be noted that they were directly compared to the previously reported METs values obtained by direct measurements such as oxygen intake. However, because the physical activity meter used in the present study has already been verified for consistency with oxygen consumption and METs estimated from the accelerometer the usefulness of the data is considered to be ensured. Second, the results of study are limited to subjects whose BMI values are within the healthy middle range. As a result, it is unclear whether taking tennis lessons would necessarily have similar results for subjects whose BMI fall outside the healthy range. Third, it is not possible to make reference to the physiological responses obtained by playing tennis, because we did not measure physiological indices such as biomarkers, which are generally used to verify whether health benefits have been obtained. Fourth, the results of this study may indicate that psychological aspects due to social interactions within the group may have influenced the amount of physical activity during the tennis lessons. However, since this study did not measure psychological aspects, it is not possible to determine their influence. Therefore, the physiological effects and psychological aspects of taking tennis lessons need to be examined in the future.

## Conclusions

We investigated the characteristics of physical activity in subjects who participated in beginner-level group tennis lessons. The physical activity intensity performed in the beginner-level tennis lessons averaged 3.37 METs, which is lower than in competitive-level tennis matches reported in previous studies. On the other hand, the short-bout rest which was 90 and 120 s, was rarely performed during the tennis lesson (7% and 4%, respectively). This was a different characteristic of the tennis match reported in previous studies and a particular feature of group tennis lessons. Taking part in tennis lessons increased the average daily physical activity intensity and resulted in more steps taken. Furthermore, in 92% of subjects, the vigorous-intensity physical activity duration increased on the days attending the tennis lesson compared to the days without the tennis lesson. In addition, 76% of subjects performed vigorous-intensity physical activity more than 1.5 times longer duration on the days attending the tennis lessons than the days without the tennis lessons. Therefore, it is suggested that participating in beginner-level tennis lessons facilitates increased duration of moderate- and vigorous-intensity physical activity compared to general daily activities and may contribute to meeting the recommended physical activity levels for promotion of health benefits.

## Methods

### Ethics statements

The study design and protocol were approved by the Observation Research Ethics Review Committee of Osaka University Hospital (code: 19537). Informed consent was obtained from all subjects in writing and verbally. The study followed the ethical recommendations for human studies, as suggested by the Declaration of Helsinki. All methods were performed in accordance with the relevant guidelines and regulations.

### Data collection

Data were collected from May 2021 to June 2021. In this study, we hypothesized that playing tennis would increase mean daily physical activity intensity by 0.2 to 0.6 and used G*-Power^27^ to detect sample size. We planned the participants and the number of days of implementation that would yield the largest sample size (84 days). The subjects were 26 adults (age, 55.6 ± 14.04 years) participating in group lessons at tennis school at the beginner level. The tennis school investigated in the present study provided 6 different classes divided based on the participant’s performance level of tennis. The 6 classes included introductory, beginner, beginner-intermediate, intermediate, advanced, and tournament classes. Those classifications were decided by the technical coach of the tennis school by observing the participant’s play during the lesson. The subjects of the present study were all classified to the beginner level. The subjects’ BMI fell within the healthy middle range, comprising a healthy population without any overweight or underweight individuals (BMI, 21.2 ± 3.07 kg/m^2^). The OMRON Active Style Pro (ASP) HJA-750C (Omron Healthcare, Kyoto, Japan) (hereinafter, referred to as “wearable device”) was used to measure physical activity, and estimates physical activity intensity in metabolic equivalents (METs) every 10 or 60 s based on the total acceleration measured by the built-in three axis accelerometer. This wearable device records tri-axial (x-, y-, and z-axis) accelerations at a measurement frequency of 32 Hz with a resolution of 3 mG. The 750C also directly predicts METs using a multiple regression model based on 12 major physical activities (seven physical activities and five lifestyle activities)^28^. A proprietary algorithm was used, and the validity of the estimated METs criteria was confirmed by measurements using the Douglas Backs technique^28^. Subjects were instructed to wear the wearable device, attached to their pants around the waist with a clip, for at least 10 h per day (excluding sleeping and bathing), and a total of six tennis lessons of 80 min per lesson were measured. In addition, physical activity during daily life was measured for four weeks, including tennis lessons.

### Data analysis

The inclusion criterion for the data analysis was that the wearable device was worn for at least 10 h per day for at least four days in a week. Valid data were categorized into days when performing or not performing tennis lessons. The structure of an 80 min tennis lesson conducted at the target tennis school consists of a 10 min warm-up, a 20 min stroke rally, a 40 min rotation drill, and a 10 min match practice. This study has been conducted in the structure of the regular tennis school lesson regardless of the study. The data measured every 10 s with a high temporal resolution were used to analyze the 80 min tennis lessons. METs were categorized into sedentary behavior (SB, < 1.5 METs), light physical activity (LPA, 1.5–2.9 METs), moderate physical activity (MPA, 3–5.9 METs), and vigorous physical activity (VPA, > 6 METs), following a previously reported definition^29,30^. The mean intensity of physical activity during daily tennis lessons (METs), physical activity intensity (METs/day), number of steps (steps/day), and duration of physical activity per day (min/day) for each physical activity intensity were obtained.

To evaluate rest periods during the tennis lessons, 90 and 120 s short-bout rests were obtained, which are the same rest periods as between the court changes and sets during a tennis match. Data processing followed previous studies^31^, and one short-bout rest was counted when all physical activity intensities for each 10-s frame were less than 2.9 METs per unit time for 90 and 120 consecutive seconds. The total number of short rests per tennis lesson was counted, and the percentage of short rests per tennis lesson was calculated by dividing the total number of short rests by the duration of the lesson. The mean percentage of short rests per lesson was then determined for 90 and 120 s, respectively.

To investigate the effect of the tennis lesson on the mean daily physical activity intensity, the following data were used: (1) physical activity intensity on the day of the tennis lesson (TL) (incl TL); (2) daily physical activity intensity excluding the time during the tennis lesson (excluding TL); and (3) daily physical activity intensity excluding the time during the tennis lesson and the travel time (TT) from the day of the performed tennis lesson (excluding TL + TT). The travel time was assumed to be 1 h each day, and the data for the day of the performed tennis lesson were analyzed after removing the activities 1 h before and after the tennis lesson.

Repeated measures analysis of variance were conducted to examine differences in physical activity intensity during daily tennis lessons and between conditions on the day of the tennis lessons. When differences between conditions were found, multiple comparisons were made using the Tukey–Kramer method. In addition, a paired t-test was used to identify differences in mean physical activity intensity and the number of steps taken on the days of performed and non-performed tennis lessons. Statistical analysis was performed using Python (Python version 3.10.1, PythonTM, Python Software Foundation, Wilmington, DE), and *p* < 0.05 were considered statistically significant.

## Data Availability

The dataset generated and analyzed during the current study are available from the corresponding author on reasonable request.
